# Making climate information services accessible to communities: What can we learn from environmental risk communication research?

**DOI:** 10.1016/j.uclim.2019.100537

**Published:** 2020-03

**Authors:** Leslie Mabon

**Affiliations:** a School of Applied Social Studies, Robert Gordon University, United Kingdom; b Scottish Association for Marine Science, United Kingdom

**Keywords:** Climate information services, Japan, Risk communication, Risk governance, Social dimensions of climate change, Urban climate change

## Abstract

This paper evaluates the role of socio-cultural issues in developing climate information services that are accessible and engaging to urban communities. Two public-facing city-level climate information provision initiatives in Japan are evaluated in light of theory in environmental risk communication. The first case is Fukuoka City, Kyushu, in particular increased flood and heat risk. The second case is Tomakomai City, Hokkaido, particularly municipal data provision on potential localised climate risks related to marine environmental change. Evaluation is undertaken through in-depth interviews with local-level actors (policymakers, scientists, NGOs, citizens), and field observation in each location. The paper argues that at a stage where principles and best practices on climate information service provision are still emerging, it is crucial to avoid assumptions about what communities will want to know about climate risks. The paper hence proposes principles for more appropriate climate risk communication. These include (a) identifying which institutions citizens look to for information on local weather and climate; (b) acknowledging that publics can, in appropriate contexts, be able and willing to engage with complex information on urban climate risk; and (c) considering how data-driven information services fit with the more informal ways in which people can experience environmental change.

## 1 Introduction

This paper clarifies issues around developing effective urban climate information services, through analysis of the cases of Fukuoka and Tomakomai cities in Japan. For the purposes of this paper, climate information services are understood in accordance with the definition of Visbeck (2008: 2), as “assessment and forecasting capability that gives public and private decision makers worldwide the best possible information on likely climatic developments from months to many decades.” Hewitt et al. (2012) continue that the objective of climate services is making society resilient to climate-related hazards. Accordingly, this paper focuses on climate information services intended for members of the public and stakeholders with less techno-scientific information and involvement in policy-making processes – which hereafter are termed ‘public-facing’ climate information services.

Along with increasing interest in cities as sites for both problems and solutions relating to climate change (Bai et al., 2018), there is rising awareness that engagement with and participation of society at large is critical if actions intended to reduce climate risk are to take root (Romero-Lankao et al., 2018). In particular, it is recognised that attaining the goal of helping publics become ‘resilient’ to environmental change in the way climate information services envision (e.g. Hewitt et al., 2012; Georgeson et al., 2017) requires more than simply providing society with ever more amounts of techno-scientific data (Chan et al., 2018). Rather, building publics' climate resilience requires attention to issues of trust in ‘experts’ and policy-makers, and perceptions of fairness in decision-making processes based on techno-scientific evidence (Mabon and Shih, 2018a; Matin et al., 2018). Indeed, recent thought on the social dimensions of climate information services argues for going beyond a linear model of information provision from research to practice and instead thinking in terms of developing relationships with users (McNie, 2013); and for bearing in mind the social, cultural and political context when working with climate data so as to develop more effective communication (Bronnimann and Wintzer, 2018).

The purpose of this paper is therefore to evaluate principles for engaging the public at large on urban climate information services, in a manner that balances the need to provide information on real and harmful phenomena with the acknowledgement that the way in which the underpinning scientific data is interpreted or utilised is not necessarily objective or value-neutral. To do so, the paper draws on the extensive body of literature into the social dimensions of environmental risk and recent thinking on environmental risk communication. This is then used to guide evaluation of public-facing climate information services in two Japanese cities with very different climates and risks from climate change – humid subtropical Fukuoka City in Kyushu, and humid continental Tomakomai City in Hokkaido.

## 2 Overview of literature

### 2.1 Social science understandings of risk

The way in which science and scientific knowledge has been considered within society has shifted over time. ‘Public understanding of science’ approaches were closely aligned with ‘information deficit’ models, viewing society at large as passive recipients who needed to be provided with techno-scientific information in order to make the ‘right’ decisions (Wynne, 2006). Over the 1990s and 2000s however, emphasis shifted towards ideas of ‘upstream engagement’ (e.g. Rogers-Hayden et al., 2007), which advocated earlier engagement with publics on issues of significant technical, scientific and ethical complexity in order to clarify concerns and information/knowledge requirements. This in turn has been supplemented by a range of approaches including co-production (Jasanoff, 2004); ‘opening up’ (Stirling, 2008), and ‘heating up and cooling down’ (Sundqvist, 2014). Whilst different, these approaches share a common concern with the idea of scientific knowledge – and discussions on subsequent courses of action – being produced through dialogue between a range of sectors in society, encompassing not only ‘experts’ but also policymakers, civil society organisations and citizens.

Alongside this, there has been concomitant development in understanding how risks are perceived by society and how they can be responded to. This matters in a climate information services context because at base, the provision of climate information is intended to help citizens and society at large understand and respond to risks (e.g. heat waves, floods, extreme weather events) which arise as a result of a changing climate (Visbeck, 2008). In a social science context, risk is widely understood to be a social construct. This does not mean ‘anything goes’ or that a changing climate will not have real effects, rather that the way in which people understand and respond to risk is informed by a breadth of social and cultural factors which shape what they perceive as an acceptable or appropriate level of risk (Bradbury, 1989; Douglas, 1992).

To give just a few examples from social science research into understandings of risk associated with climate change, publics' views on risk and on an appropriate course of action may be informed by: trust in the competence of the people and institutions who are assessing and communicating risk on society's behalf (Gough and Boucher, 2013); the extent to which the climate risk in question receives political and media attention and is related to wider political issues (Mabon and Shih, 2018a); fit between personal identity and value systems and that of the institution conveying information about the risk (Milnes and Haney, 2017); and gender roles and the ability of women within societies to be able to access and receive practical information (McOmber et al., 2013).

In short, in the context of urban climate information services, there is ample evidence to suggest that providing ‘more’ and ‘better’ information is in itself unlikely to be effective if attention is not paid to the wider social and cultural factors which drive how the information provided is received. How these complex factors may be reconciled within the development of urban climate information services is the subject of the following sub-section.

### 2.2 Risk governance and risk communication

Continuing the thread of risk being socially mediated as discussed above, van Asselt and Renn (2011) hold that not all risks can be easily calculated into probability versus effect, and that individuals and institutions – both public and private – all need to deal with uncertainties and indeterminacies around risks. It is through processes of risk governance (Renn, 2008) that these competing pressures and concerns around what constitutes an appropriate response to risk are managed, and collective decisions around complex social issues are taken.

This idea of risk having to be ‘governed’ is significant in the context of climate information services if we return to the underlying purpose of climate information services being to allow society to make the best decisions possible in response to climate risks. Indeed, McNie (2013) argues effective development of climate information services necessitates going beyond a linear model of communicating research to decision makers, to consider a much wider set of factors including identification of user needs, translating and sharing knowledge, developing people's capacity to understand complex data, and building strong social connections and leadership. This resonates strongly with the understanding that risk communication ought to be about not only information provision and correction of misunderstandings (Bradbury, 1989; Arvai, 2014), but rather a much more dialogic process on what kind of information and data is required, by whom and in what format. Kasperson (2014) proposes four key principles for this more nuanced form of risk communication in response to complex situations: (a) better-sustained and better-funded programmes; (b) wider consideration of risk issues in values and lifestyle structures; (c) identification of the uncertainties that matter most to publics and development of appropriate communication; and (d) avoiding assumption about the kind of information that will be required by publics, and finding ways to empower risk-bearers. Given that response to climate change in cities is widely considered a socially and ecologically complex issue (e.g. Tyler and Moench, 2012; Bahadur and Tanner, 2014), there is again therefore good reason to suggest that in order to most effectively enable publics to manage climate risks, urban climate information services ought to be developed under this more dialogic ethos of risk communication. The following section attempts to spell out what the specific challenges are in this regard for climate change and climate information in an *urban* setting.

### 2.3 Resilient cities, climate information, and risk communication

The aim of making cities and the people within them resilient to a changing climate – that is, able to retain their core functions when faced with external shocks (Connolly, 2018) – has significant political currency. This is evidenced by, for example, the mentioning of resilience in Sustainable Development Goal 11; and through the prominence of resilience thinking in high-profile urban fora such as ICLEI's *Resilient Cities* series and the Rockefeller Institute's *100 Resilient Cities* programme. Climate information services have been described as key to attaining this resilience to climate-related hazards. Hewitt et al. (2012: 831) justify climate information services as a way to “improve society's resilience to climate-related hazards and better manage the risks and opportunities arising from climate variability and climate change.” Georgeson et al. (2017:1) too state that climate information services have an important function for building climate resilience.

On one hand, resilience can be understood in a more traditional engineering sense as the ability to understand complex systems and act before failures and harm occur (Hollnagel et al., 2006). In this way, provision of climate information has an important role to play in making urban citizens ‘resilient’ by helping them to understand changes and risks in the environment around them in a way that will reduce harm from very real and tangible effects of climate change such as heatwaves, flooding and extreme weather events (Mabon and Shih, 2018b). At the same time, however, it is important to acknowledge more critical takes on ‘resilient’ cities and citizens (e.g. Connolly, 2018; Matin et al., 2018), which argue that strategies to make citizens resilient to external shocks and stresses (and therefore able to cope by themselves) can divert attention away from the underlying social processes (e.g. unequal access to education, climate risk reduction measures disproportionately accruing towards more affluent communities) that mean some people are at greater risk of harm from climate change than others in the first instance. Extending this into the realm of urban climate information services, if such information services are to play a part in this more holistic understanding of a resilient city it is hence important that development of urban climate information services do not inadvertently repeat or reinforce the processes which make some people more vulnerable to a changing climate – and less able to access information and knowledge which may help them to reduce their exposure to risk. In practice, this means there is a need to ensure that urban climate services are targeted towards the areas and people who are most vulnerable and require this information the most. Building on 2.1, 2.2 above, it is particularly important that attention is paid to the socio-cultural dimensions of risk and risk communication, and that processes of risk governance are drawn on in order to ensure urban climate information services are responsive to the needs and requirements of vulnerable populations. Given the discussion above about issues such as trust, identity and values, risk governance for urban climate information services for publics may encompass not only the type of information provided, but also issues such as in what format the data is presented, by whom, and where.

This paper hence sketches out how some of these complexities can be addressed in practice in a particular locale to attain ‘risk communication’ through climate information for resilient cities. This is undertaken through evaluation of two Japanese contexts with different climate risks and public-facing climate information service contexts – Fukuoka and Tomakomai. On one hand, Fukuoka is a humid subtropical city which mainly faces risks from heat and flooding, yet where there is a long tradition of research into the urban climate and where there is well-developed information provision for publics on climate-related risks (see Section 4). Tomakomai, meanwhile, is a smaller humid continental city, where understanding of the localised effects of climate change – and therefore also of the strategies and platforms to communicate climate risk to citizens – is still emerging. Moreover, in Tomakomai, issues around public-facing risk communication for climate information also touch on mitigation, in that the city hosts Japan’s first large-scale demonstration of sub-sea carbon dioxide storage (see Section 5). These two cities hence give very different contexts in which to assess the socio-cultural aspects of public-facing climate information services.

## 3 Method

**Fig. 1:**
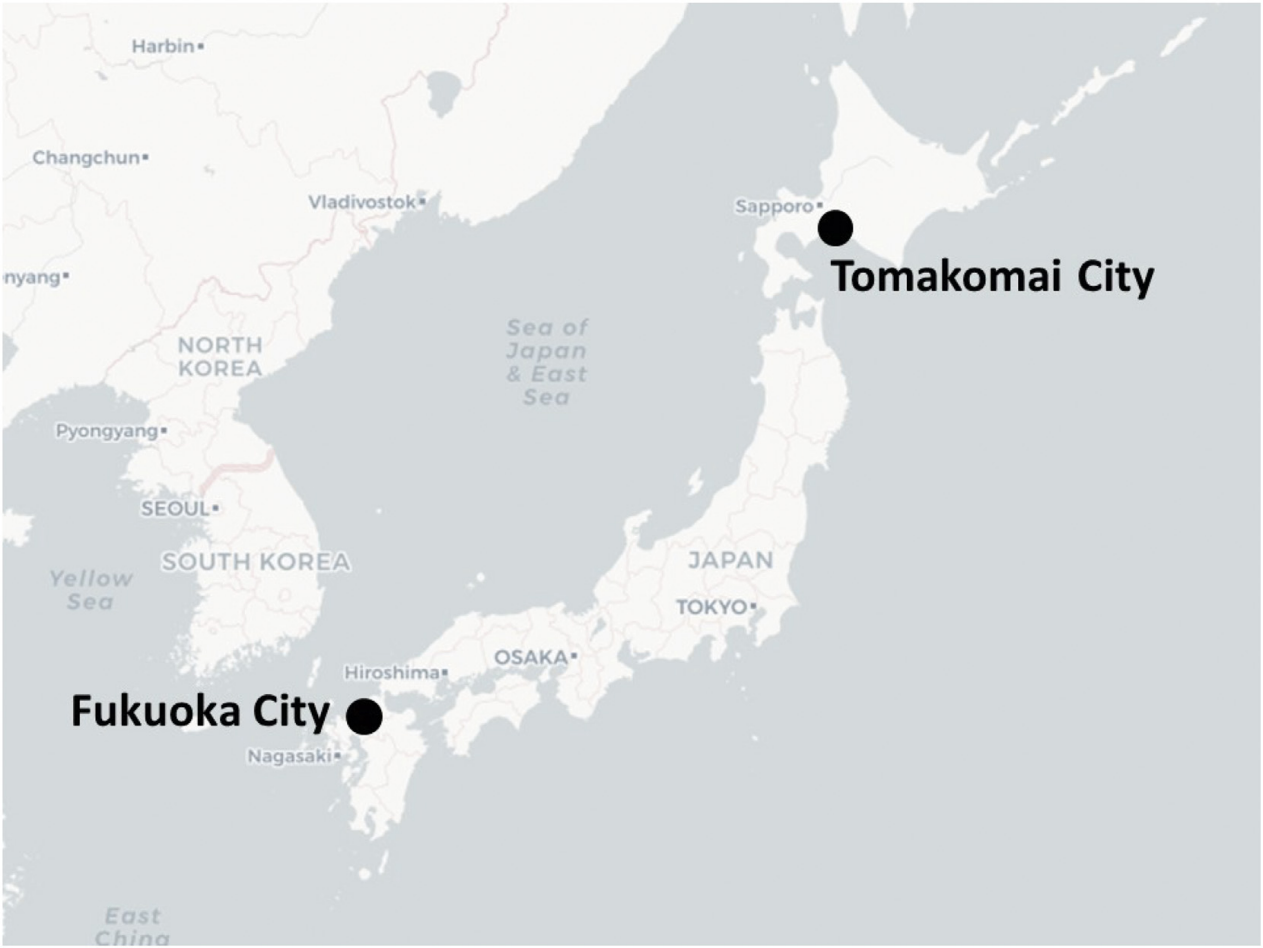
Location of Fukuoka City and Tomakomai City within Japan (adapted from map tiles by Stamen Design, under CC BY 3.0. Data by CartoDB and OpenStreetMap, under ODbL).

This paper draws on data from two larger projects which look, in broad terms, into the human dimensions of climate change in the case study areas and in particular how to govern risks associated with environmental change. Specifically, these are a study into the governance of urban heat and climate adaptation through the lived environment in Fukuoka, Japan; and a study into climate change mitigation and the role of carbon dioxide capture and storage within this in Tomakomai, Japan (see Fig. 1). For a fuller overview of these projects including overarching research design and methodology, see Mabon et al., 2019 and Mabon et al., 2017 respectively. In both projects, the availability and quality of knowledge and data which could be drawn on to plan responses to climate risk formed a central component of wider discussions on environmental governance. This paper therefore draws on the elements of the data gleaned from these projects which illustrate opportunities and challenges for developing and deploying climate information services in an urban setting.

### 3.1 In-depth interviews

The paper is based mainly on the findings of in-depth interviews undertaken with key risk-managers and risk-bearers in each city context. The purpose of analysing the interviews is to (a) understand some of the strategies being used to engage with citizens and stakeholders on local climate changes; and (b) get a sense of the social and cultural context around which provision of climate information happens in each locale. Whilst the data for each project was not collected primarily to look at urban climate information services, the datasets for both cities nevertheless yield insights into how people in each location engage with and understand data relating to climate change. The topic guides used for the interviews in each city are included as Supplementary Data.

For both the underpinning research and also the aims of this paper, sampling was intended to encompass both those providing and creating the data for local climate change governance and associated urban climate information services (e.g. local government officials, academics and research institutes); and also those likely to access environmental data, including public-facing climate information services (e.g. citizens and stakeholders less connected with research and governance processes). Participants involved on the policy- decision-making side were identified by looking to municipal government climate change action plans in the first instance (e.g. Tomakomai City, 2013; Fukuoka City, 2016); and thereafter contacting and recruiting representatives with responsibility for local climate governance as well as researchers/research institutes listed as providing data or input to local governance strategies. Participants on the citizen/stakeholder side were focused on what we have elsewhere termed ‘informed publics’ (Mabon et al., 2014) – i.e. people who have personal interest in environment and climate issues and/or may have a stake in decisions taken about risk governance through their work, and hence are more likely to be able to provide meaningful input and rich insight into the risk communication challenges for climate information services. Sampling and recruitment for this side was carried out through a web and media search of local opinion-shapers, and also through a snowball-sampling approach to locate relevant expertise (Atkinson and Flint, 2001). Relevant interviews extracted from the larger project data datasets and used to form the basis of this paper are summarised in Table 1.

**Table 1:** Overview of interviewees.

Interviewed organisation/institution	Sector	Relation to local urban climate information services
Fukuoka		
Fukuoka City Government Environment Division	Municipal government	Provider of public-facing climate change communication in Fukuoka City
Fukuoka City Government Green City Promotion Department	Municipal government	Provider of additional public-facing climate change information in Fukuoka City; planning for flood- and heat risk mitigation
Fukuoka Prefecture Environment Division	Regional government	Provider of public-facing climate change communication across wider Fukuoka region
Fukuoka Center for Climate Change Actions	Civil society	Citizen engagement on climate change; training of community climate volunteers
Kyushu Environmental Evaluation Association	Academia and research	Undertake research into urban thermal environments in Fukuoka City, feed into city plan; also support FCCCA
Academic involved in Fukuoka city climate change action plan expert committee	Academia and research	Undertake research into urban thermal environments in Fukuoka city, feed into city plan; also communication via teaching
Academic involved in regional adaptation planning in Japan	Academia and research	Undertake collaborative research with regional governments into climate adaptation planning including risk communication
Environment and climate consultancy	Private sector	Assess public awareness and information requirements via market research

Tomakomai		
Tomakomai City Government Industrial Location Promotion Division	Municipal government	Provider of public-facing climate change information; and engagement of citizens and key stakeholders via Tomakomai CCS Demonstration Project
Japan CCS Company	Private sector	Major opinion-shaper and source of information for citizens on climate change in Tomakomai via Tomakomai CCS Demonstration Project
Tomakomai Port Authority	Public sector	Development of future business and management strategies based on climate information
Hokkaido Government Climate Change Mitigation Group	Regional government	Provider of public-facing climate change information at regional level; development of regional climate change action plan
Hokkaido Government Environment and Energy Group	Regional government	Provider of public-facing climate change information at regional level; citizen communication on climate issues and actions
Tomakomai Fisheries Cooperative Association	Private sector	Main recipient of climate change information via concerns about effects of climate (and also mitigation strategies e.g. CCS) on fish stocks
Hokkaido Center for Climate Change Actions	Civil society	Citizen engagement on climate change via information communication
Academics specialising in geology for subsea activity	Academia and research	Engagement with fishers on environmental change and mitigation strategies (e.g. acidity monitoring)
Urban planner in analogous city context, Hokkaido	Municipal government	Responsibility for considering urban planning actions in response to climate information and predictions
Tomakomai City Chamber of Commerce	Private sector	Consideration of risks and opportunities to local business as a result of predicted climate effects on Tomakomai City
Academic at Tomakomai Komazawa University	Academia and research	Consideration of risks and opportunities to local economy on basis of climate information, via specialisation in carbon accounting

As far as sample size is concerned, in qualitative research of this nature the richness of the data provided can count for more than the size of sample and number of people interviewed (e.g. Marshall, 1996). Given the complexity of the topic, a smaller sample of people with good knowledge was hence deemed to be more valuable for in-depth discussion on issues and challenges around urban climate information services than a wider-ranging but less knowledgeable sample.

### 3.2 Field observation and review of existing public-facing climate information services

Interviews were supported by field observation of exhibitions, displays and events related to public-facing climate information services in both Fukuoka and Tomakomai; and by collation and review of public-facing climate information service-related material available in both cities such as websites, information leaflets and display boards (an inventory of the main public-facing climate information services which exist for each city and were reviewed for this study is provided in Section 4.2 for Fukuoka; and Section 5.2 for Tomakomai). This observation and review provided additional contextual information on how climate information is developed with and for citizens and stakeholders. Descriptive observations were recorded via note-taking and photography (Blomberg et al., 1993), and used to support the insights from the interviews. Public lectures on climate information and climate risk hosted in Fukuoka (Fukuoka Center for Climate Change Actions) and Tomakomai (Tomakomai Komazawa University) were attended; and information exhibitions on climate risk preparation in both cities were also visited.

### 3.3 Analysis

As the interview data on which this paper is based was originally collected for separate studies, this paper utilises a form of secondary qualitative data analysis (Bryman, 2012), whereby existing qualitative data is re-analysed for a different purpose. Given that this study also combines new insights from textual and observational data and is concerned primarily with addressing an empirical question – specifically, evaluating principles for public engagement with climate information services – a qualitative content analysis was deemed appropriate. Qualitative content analysis, broadly defined, entails formatting qualitative material into categories or themes that answer the research questions (Cho and Lee, 2014). Cho and Lee explain that qualitative content analysis of this nature is appropriate for situations where, as per this study, the aim is to understand a social phenomenon and where data is sampled from a range of sources. Accordingly, insights from the interview data, review of public-facing climate information services and field observations for each city were noted under the following categories: (a) current and recent relationship with climate and environmental risks in the city; (b) future climate risks perceived as being faced by the city; (c) available sources of local environmental data; (d) perceived uncertainties or limitations associated with available local environmental data; (e) institutions and individuals within the city trusted to provide information on climate risks; and (f) wider socio-cultural context within which climate change is happening. For an empirical study of this nature, organising the available data in this way helped to meet the paper’s objective of understanding the socio-cultural context around urban climate information services by clarifying not only what the communication strategies for climate information services employed by each city are; but also how this information is being received by local society. This analytical framework is also in keeping with the theoretical basis of the paper (Section 2), which understands risks as being perceived and responded to differently by different people depending on their social and cultural context.

## 4 Case study 1: Fukuoka

### 4.1 Overview

**Fig. 2:**
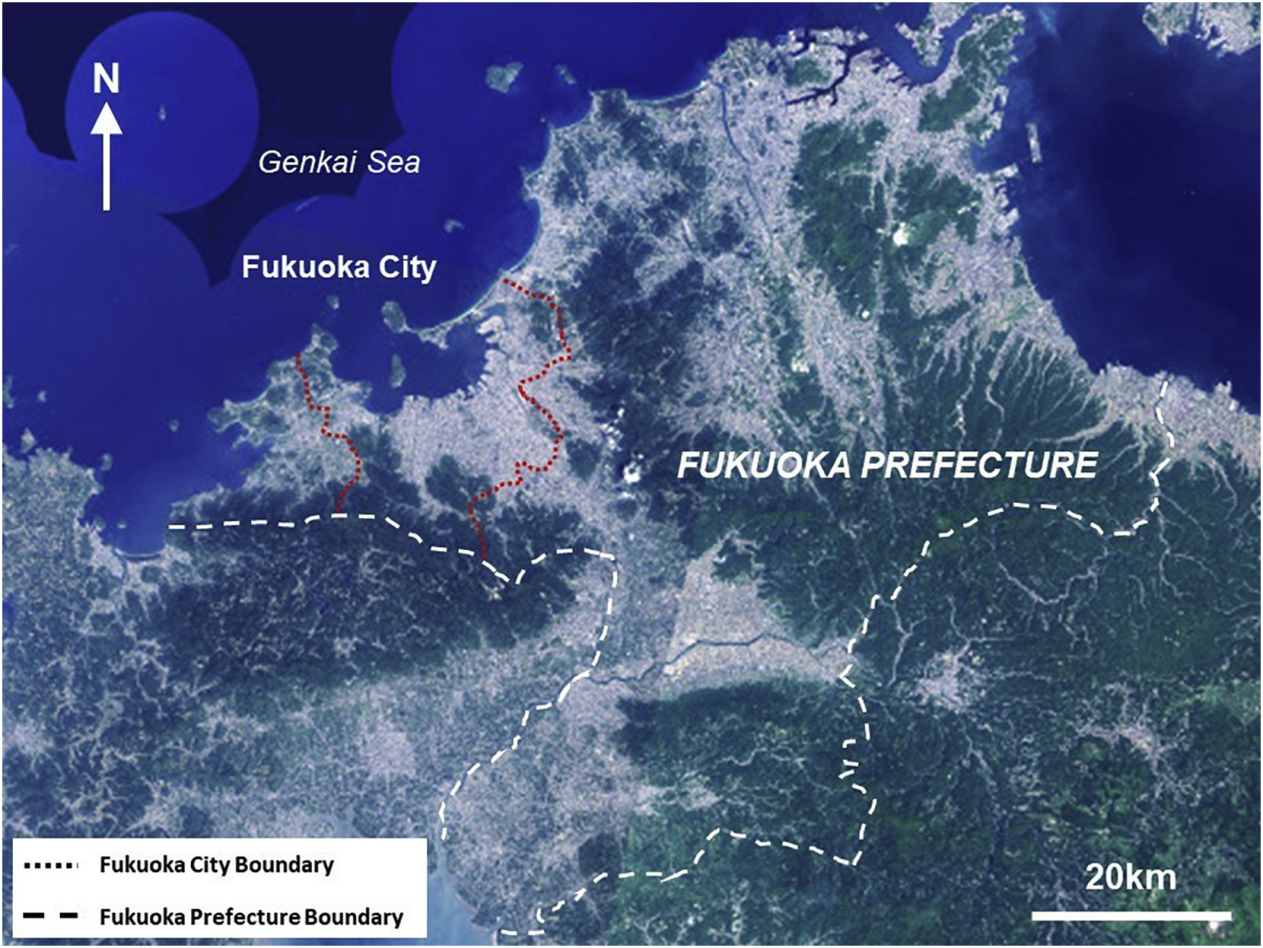
Location of Fukuoka City within Fukuoka Prefecture (source: adapted from Geospatial Information Authority of Japan, 2018).

Fukuoka is located in north Kyushu, the southern-most of Japan's four main islands. It is bordered by Hakata Bay (which opens into the Genkai Sea) to the north, and by the Sangun and Sefuri Mountain Ranges to the east and south-west respectively (see Fig. 2). Fukuoka City is built on the Fukuoka Plain, with a population of approximately 1.5 million people in 2018, over an area of 340 km2 (Fukuoka City, 2017; Mabon et al., 2019). Fukuoka City has a humid subtropical climate, with the highest temperatures reaching around 37 °C in the months of July and August and an average of 1612 mm of rainfall annually (Fukuoka City, 2017).

Fukuoka has the biggest population growth of any major city in Japan, with 7.1% growth between 2010 and 2017 (Fukuoka Asian Urban Research Center, 2018). Notably, in 2016 people over 65 – a group often considered vulnerable to climate risks – made up 21.4% of the city population (Fukuoka City, 2017). Fukuoka's economy is supported primarily by service industries, followed by wholesale and real estate (Fukuoka Asian Urban Research Center, 2018).

### 4.2 Climate risks and main public-facing climate information services

Average annual air temperatures in Fukuoka Prefecture increased by 2.54 °C between 1898 and 2017, compared to 1.69 °C for the wider Kyushu and Yamaguchi area and 1.19 °C for Japan over the same period (Fukuoka District Meteorological Observatory, 2017). Average air temperatures in Fukuoka Prefecture are predicted to increase a further 2.9 °C by 2100, with 18 more extremely hot days (over 35 °C) and 42 more hot days (over 30 °C) per year predicted by 2100 (Fukuoka District Meteorological Observatory, 2014). Fukuoka has hence experienced above-average warming thus far, and is likely to continue to feel the effects of climate change into the future (Mabon et al., 2019). Fukuoka City's Climate Change Countermeasures Action Plan accordingly identifies five key climate risks requiring adaptation actions: (a) natural hazards from heavy rainfall and flooding; (b) pressure on water resources; (c) health risks from increased heat; (d) biodiversity loss; and (e) effects on agricultural produce (Fukuoka City, 2016).

Table 2 summarises the main public-facing climate information services in Fukuoka City. Existing research at the climate risk and governance interface for Fukuoka includes: production of an urban climate atlas focusing on urban heat islands with urban climate simulation (Yoda and Katayama, 1998); analysis of slope disaster risk attributable to global warming, which fed into disaster recovery planning for Fukuoka City (Yasuhara et al., 2012); expert support for citizen-led rainwater retention initiatives (Yamashita et al., 2016); and survey-based research into citizen understandings of climate change and use of air conditioners (Chen et al., 2018).

**Table 2:** Main public-facing climate information services in Fukuoka City.

Title	Contents	Provider	Link/Citation
Fukuoka City Heatstroke information	Daily summer high temperatures; daily count of heatstroke victims with demographic details; information on heatstroke and countermeasures	Fukuoka City Government Environment Division (heat index information via Ministry of Environment)	http://heatstroke.city.fukuoka.lg.jp/
Fukuoka City social media heat alerts	Daily postings on Twitter and LINE (+ email subscription service) indicating heat alert level across different times of day, and also wider disaster prevention information e.g. heavy rainfall and evacuation alerts	Fukuoka City Government Publicity Team (data via Fukuoka City Environment Division)	https://twitter.com/Fukuokacity_pr
Information on climate change predictions for Kyushu and Yamaguchi Prefecture	Provision of regional climate data and predictions for Kyushu and Yamaguchi Prefecture based on IPCC A1B scenario	Fukuoka District Meteorological Observatory	Fukuoka District Meteorological Observatory (2017)*Information on climate change predictions for Kyushu and Yamaguchi Prefecture: Second Edition.* Fukuoka District Meteorological Observatory: Fukuoka.
Fukuoka City flood hazard map	Flood risk maps available by ward, with evacuation guidance and evacuation shelters. Both digital (downloadable PDF) and physical (hazard maps posted in neighbourhoods) versions	Fukuoka City Government Citizen Division	http://bousai.city.fukuoka.lg.jp/hazard/index.html
Fukuoka Center for Climate Change Actions (FCCCA)	Guidance, seminars, events and training on climate change adaptation and mitigation for publics, including explanation of possible effects	Kyushu Environmental Evaluation Association/Fukuoka Prefecture	https://www.ecofukuoka.jp/
Heatstroke prevention street campaign and panel exhibition	Distribution of information leaflets on heat risk in city centre with face-to-face discussion with city government staff; plus posters explaining heat risk	Fukuoka City Government Environment Division	http://heatstroke.city.fukuoka.lg.jp/torikumi/

### 4.3 Climate risk communication issues observed in Fukuoka

The first point to note from the case of Fukuoka is that the local social context is a significant explanatory factor for the availability of extensive public-facing climate information services in the current day. Fukuoka and the wider Kyushu region had negative experience with environmental pollution incidents in the 1960s and 70s, most notably Minamata Disease (where discharges of poisonous metals into Minamata Bay led to people being born with severe birth defects) and air pollution in Kitakyushu City. This led at least in part to the emergence of a number of academics and research institutions with a motivation to collect environmental data in order to ensure public health and well-being (interview with Fukuoka Prefecture Environment Division). Indeed, the Kyushu Environmental Evaluation Association was established in the early 1970s by Kyushu University academics with the explicit intention of providing independent data on water pollution, before subsequently expanding to issues of air quality, urban environments and latterly climate change (Kyushu Environmental Evaluation Association, 2018).

**Fig. 3:**
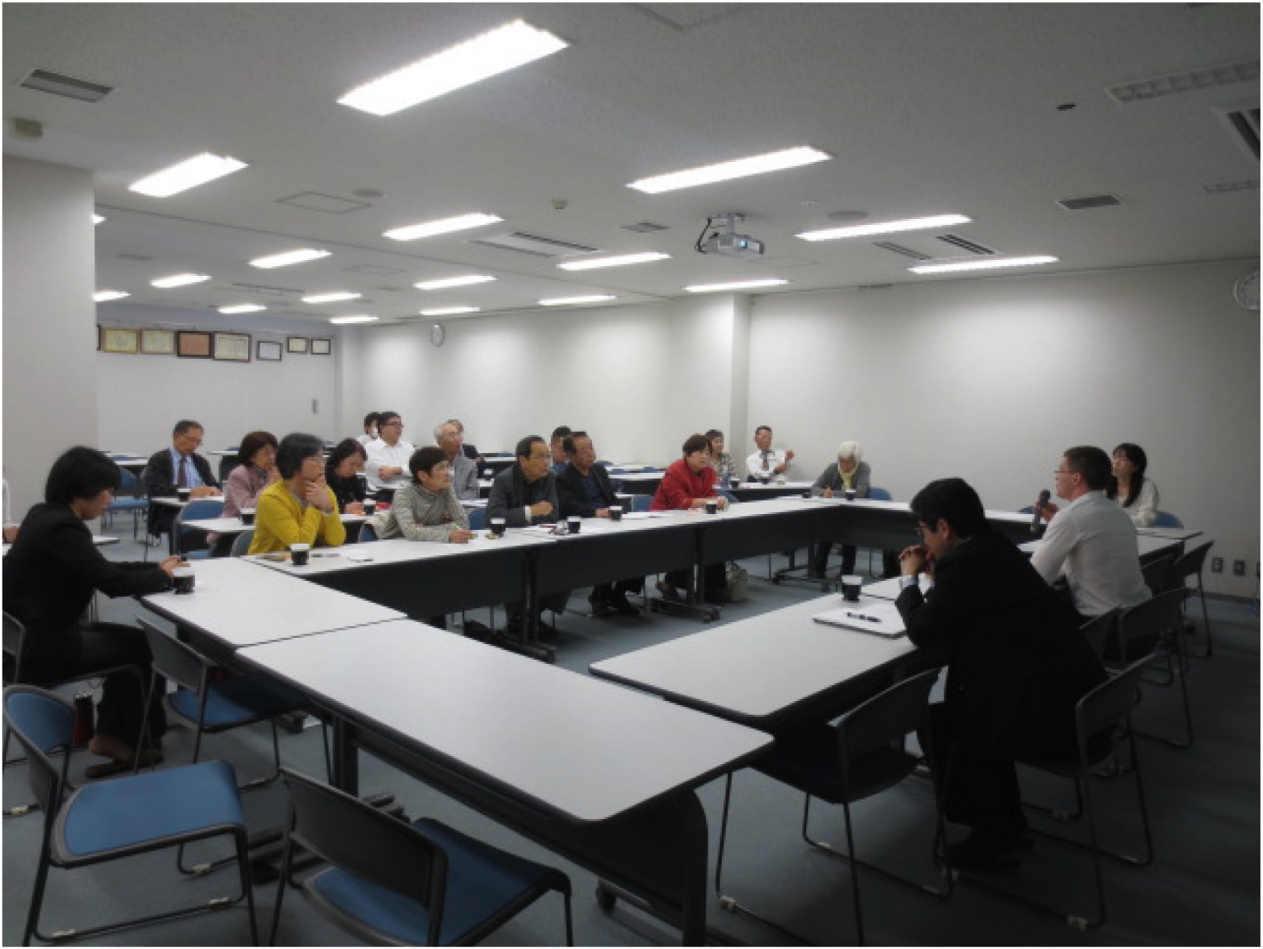
Training session at Fukuoka Center for Climate Change Actions with community climate volunteers.

Whilst such actions clearly pre-date current thinking on climate risk, this context means that Fukuoka has a tradition of local research institutions undertaking environmental science and knowledge/data provision in the public interest, who have been able to develop and adapt these competences to the urban climate context (Mabon et al., 2019). Today, for instance, the Kyushu Environmental Evaluation Association is responsible for running the Fukuoka Centre for Climate Change Action, a key source of climate information for citizens through seminars, public events and drop-in sessions (see Fig. 3) and a forum for training community-level climate volunteers to engage fellow citizens on climate responses (interview with Fukuoka Centre for Climate Change Actions). It is also worth noting that a trigger for data collection to understand urban thermal environments in central Fukuoka from the early 1990s onwards (e.g. Katayama et al., 1993) was the observation that school playgrounds in Fukuoka were hotter than the surroundings, and drive to do something in response (interview with environmental research institute). This long tradition of environmental data being collected in the public interest to maintain societal wellbeing within Fukuoka, and of the empowering of citizens through provision of knowledge of climate effects and support for community-level activities, appears to fit well with the social justice dimensions of a resilient city raised in the literature review.

**Fig. 4:**
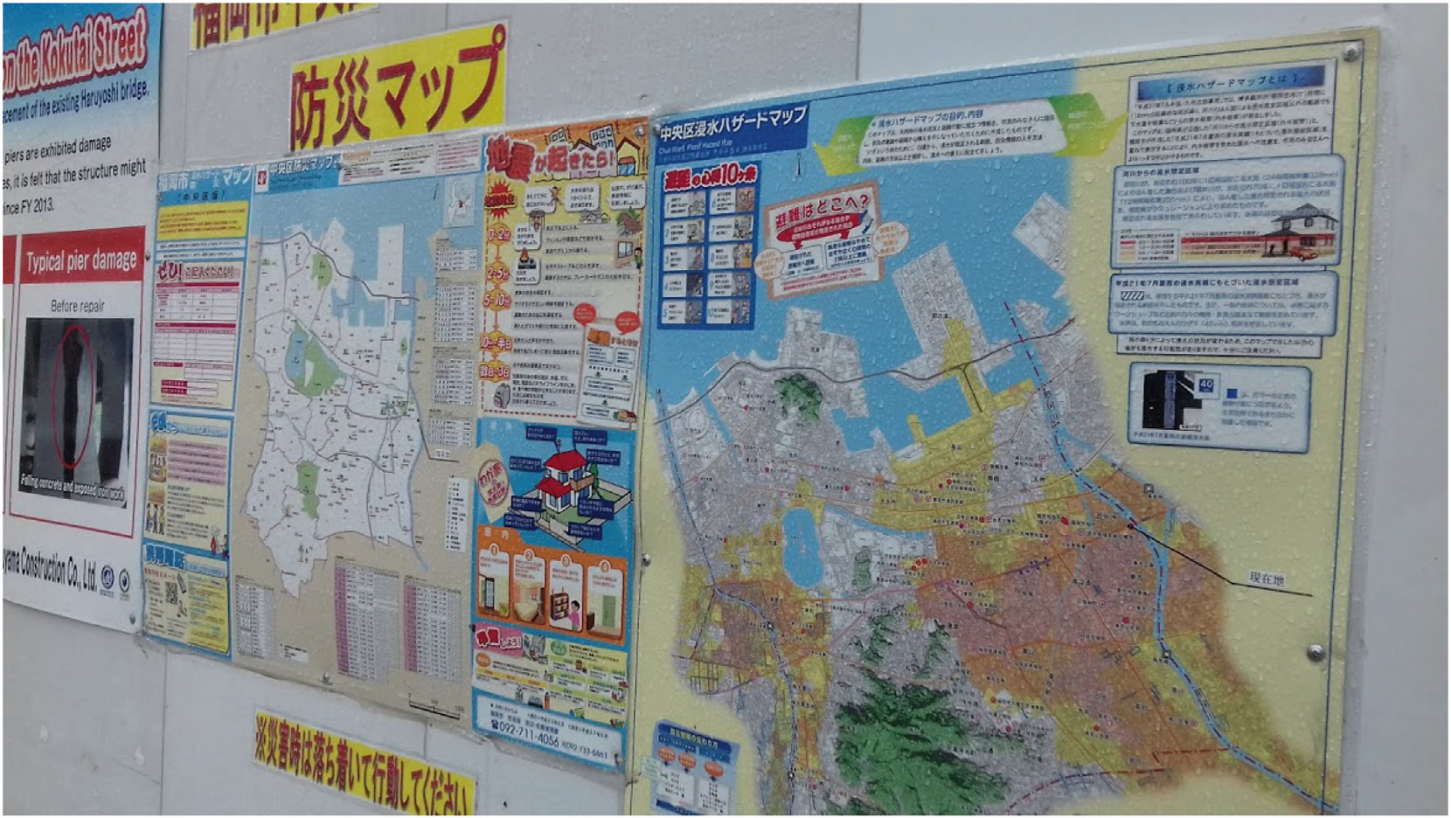
Public flood evacuation map in Chuo Ward, Fukuoka City.

However, the second point to note is that whilst Fukuoka is well-supported with institutions who can provide robust data for climate information services, the ability of this data to be shared and translated into effective and sustained communication is arguably more limited. For instance, much of the work to develop an urban climate map for Fukuoka (e.g. Yoda and Katayama, 1998) and to identify hot spots and potential cooling measures in the central Hakata-Tenjin area (e.g. Tanaka et al., 2009) has received governmental support, either directly from the national government or via the appropriate department at the municipal level. Interviewees indicated that for government-supported projects, questions around who owns and has access to data after the completion of the project can in cases make it difficult to share data with those outside the project team and/or to conduct further follow-up analysis (interview with environmental research institute). Furthermore, within the city government itself, division of responsibilities relating to climate risk between different government departments may lead to difficulty in sharing information and coordinating engagement and communication actions. For instance, the Fukuoka City Climate Change Countermeasures Action Plan and associated communication around climate change actions – including the city's heatstroke risk portal (heatstroke.city.fukuoka.lg.jp) - are the responsibility of the city's Environment Division (Fukuoka City, 2016). However, flood hazard maps (bousai.city.fukuoka.lg.jp/hazard/index.html) are the responsibility of the Disaster Prevention Section within the Citizen Division (see Fig. 4). Interviewed officials did acknowledge this was a challenge they were working to address through annual forums to connect staff working in different departments (interview with Fukuoka City Green City Promotion Division). Nonetheless, given the importance of dialogue to effective risk communication outlined in Section 2, this complexity may make it harder for citizens to engage with risk governance processes or to know where to turn for relevant information.

## 5 Case study 2: Tomakomai

### 5.1 Overview

**Fig. 5:**
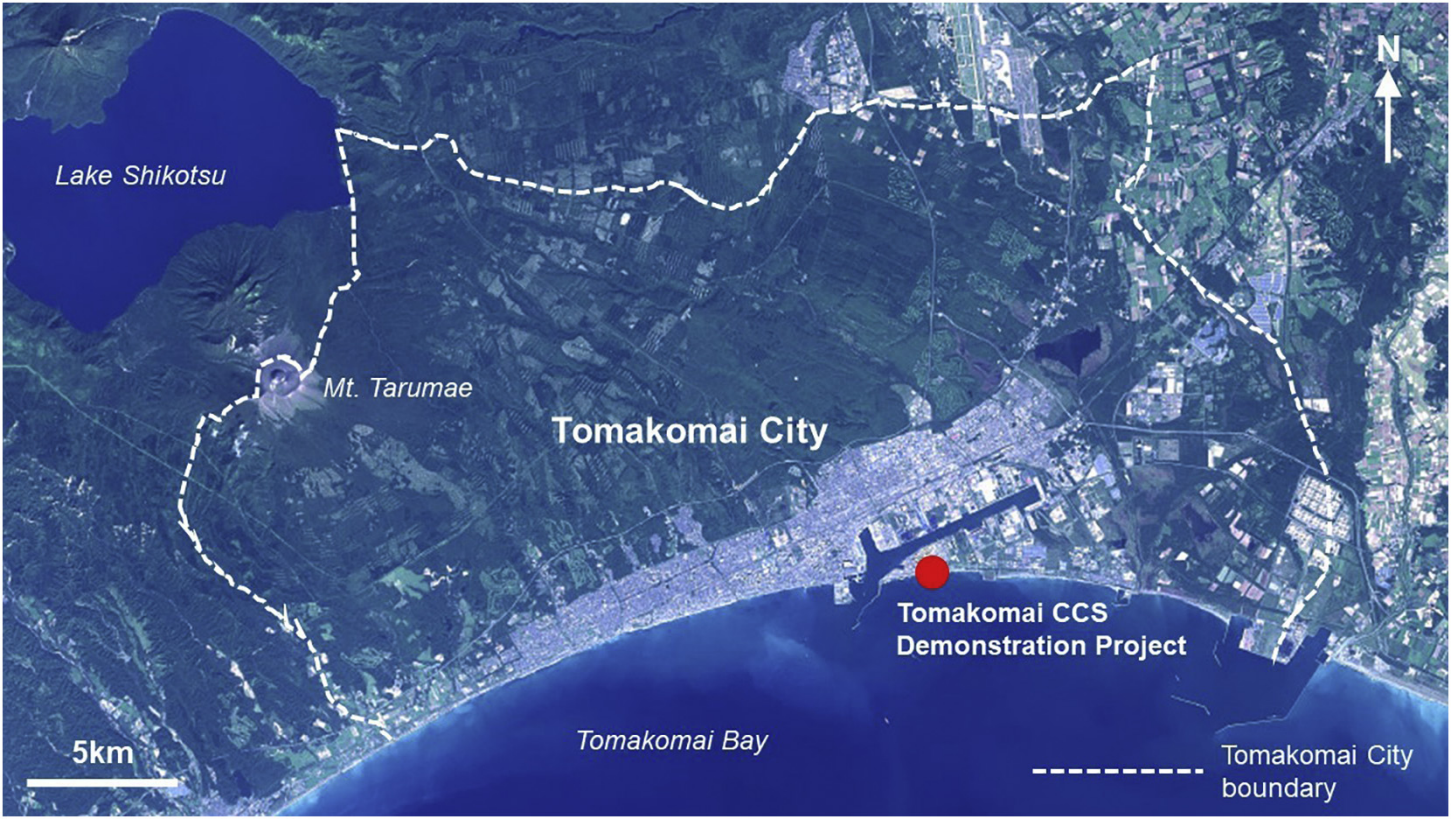
Location of Tomakomai City and key features (source: adapted from Geospatial Information Authority of Japan, 2019)

Tomakomai City is located in the south of Hokkaido, the northernmost island of Japan, within Iburi Subprefecture. It has a population of approximately 172,000 (Tomakomai City, 2013), making it the fifth-largest city in Hokkaido. The urbanised area of Tomakomai extends across a largely flat area approximately 20 km from east to west and 5 km from south to north. Tomakomai is bordered on the south by Tomakomai Bay and in turn the Pacific Ocean, and to the north-west by Mount Tarumae (see Fig. 5). The city has a humid continental climate, with average temperatures ranging from −3.8 degrees Celsius in winter to 20.3 degrees Celsius in summer (Tomakomai City, 2013). Tomakomai is reliant on carbon-intensive industries for employment and economic benefit, notably paper manufacturing, petrochemicals and shipping, however the city is also host to Japan's first large-scale demonstration of carbon dioxide capture and storage (CCS) climate mitigation technologies, injecting carbon dioxide captured from a gasification plant into geological structures under Tomakomai Bay (Mabon, 2018).

**Fig. 6:**
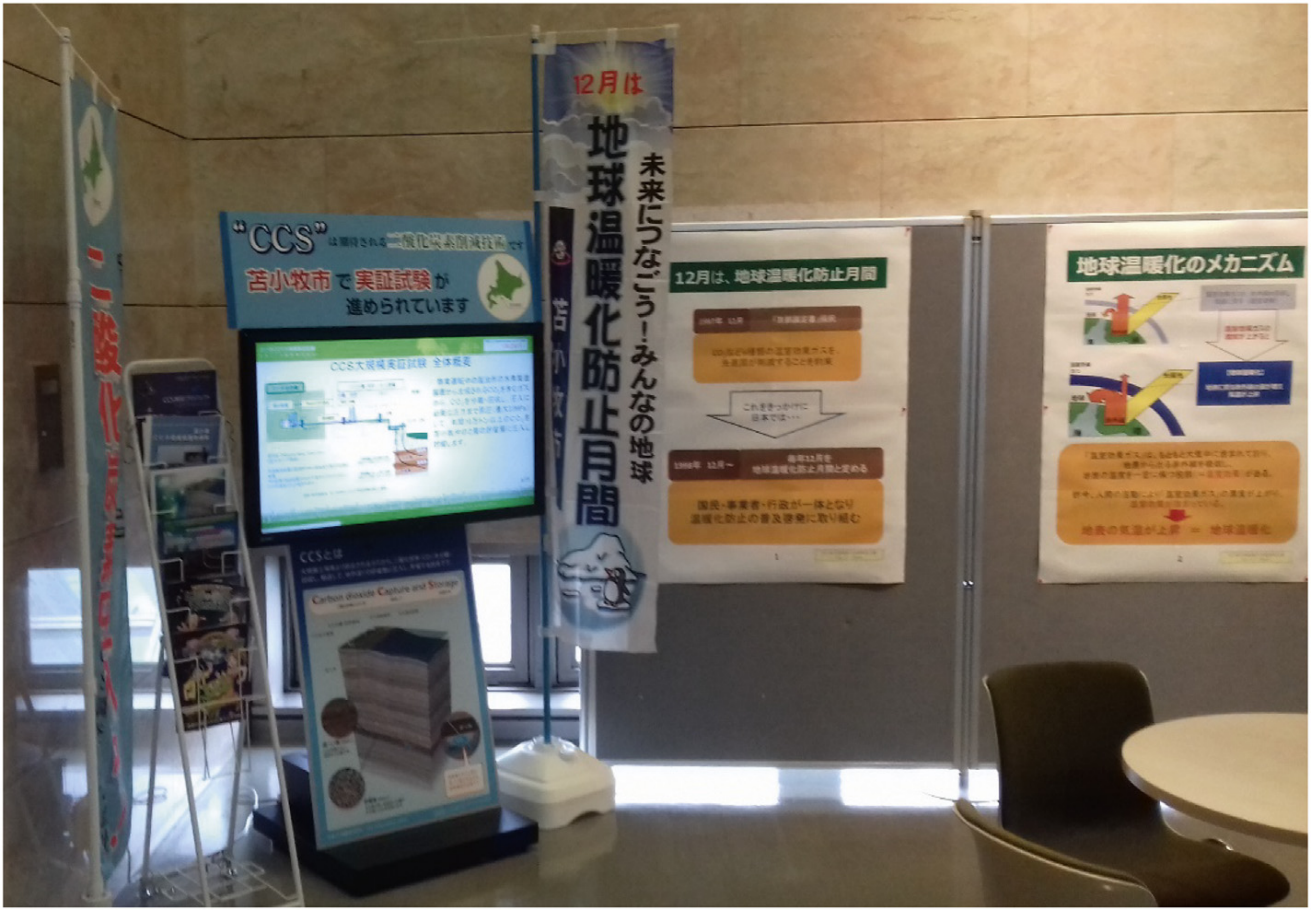
Poster exhibition on climate change effects, Tomakomai City Hall.

### 5.2 Climate risks and public-facing climate information services

Compared to Fukuoka, Tomakomai City's Local Plan for Promotion of Climate Change Countermeasures is much less specific in terms of the climate risks Tomakomai in particular is likely to face. The city's climate change plan highlights climate change effects relevant to all of Japan, namely: effects on agricultural produce; bleaching of coral reefs; decline of beech forest; and reduction in drift ice (Tomakomai City, 2013). The city's climate plan does note however, that from 1943 to 2007 the average temperature in Tomakomai increased by 1.2 degrees Celsius, above the average for all of Japan (Tomakomai City, 2013). Furthermore, Tomakomai City's Disaster Prevention Handbook lists the main natural hazards for the city as earthquakes; volcanic eruptions; and wind and flood damage and landslide risk from typhoons and heavy rain (Tomakomai City, 2018). Of these, flooding and inundation are a particular concern given the flat topography and coastal location of Tomakomai – the city's disaster prevention handbook notes much of central Tomakomai is at risk of flooding in the event of heavy rainfall or inundation (Tomakomai City, 2018).

Table 3 provides an overview of the main public-facing climate information services available which are specific to Tomakomai. Existing research at the climate risk and governance interface specific to Tomakomai is more limited than for Fukuoka, but includes: enquiry into diminishing sea ice and its potential economic benefits for Tomakomai as a port city (Kitagawa and Otsuka, 2014); and extensive enquiry into public and stakeholder engagement for the CCS demonstration project situated within the city (Suzuki et al., 2019).

**Table 3:** Main public-facing climate information services in Tomakomai.

Title	Contents	Provider	Link/Citation
Tomakomai City Local Plan for Promotion of Climate Change Counter-Measures	Monthly average, peak and minimum temperatures; atmospheric pressure; wind speed; daylight hours and snowfall 1981–2010. Also summary of climate effects for all Japan, and local CO_2_ emissions (note: updated plan to be released 2019)	Tomakomai City Government (data from Japan Meteorological Agency)	Tomakomai City (2013)*Tomakomai City Local Plan for Promotion of Climate Change Countermeasures* (in Japanese) Tomakomai City Government: Tomakomai.
Tomakomai City Disaster Prevention Handbook	Hazard maps for local areas, plus illustrated preparation and evacuation guidance	Tomakomai City Government	Tomakomai City (2013)Tomakomai City, 2018*Tomakomai City Disaster Prevention Handbook* (in Japanese) Tomkomai City Government: Tomakomai.
Tomakomai City disaster map	Online zoomable GIS hazard map, with links to disaster prevention information	Tomakomai City Government	http://www.city.tomakomai.hokkaido.jp/contents/bousaimap/
JCCS Tomakomai Monitoring	Ocean acidity data (as well as seismic and pressure information relating to CO_2_ storage site)	Japan CCS Company	http://www.jccs-tomakomai-monitoring.com/JCCS/index.php/top/
Tomakomai City Hall social media page	Extreme weather event warnings and evacuation orders; occasional information on climate change	Tomakomai City Government (updates from Crisis Management Room)	https://www.facebook.com/city.tomakomai/
Tomakomai City Hall physical exhibition space (see Fig. 6)	Poster/digital exhibition on climate change effects and actions relevant to Tomakomai City	Tomakomai City Government (data from Japan CCS Company,)	Tomakomai City Hall public exhibition space

### 5.3 Climate risk communication issues observed in Tomakomai

A first notable issue around climate risk governance and communication in Tomakomai relates to the possible effect of flagship projects or major pieces of low-carbon infrastructure in a locality on societal awareness of climate change and, by extension, on engagement with climate information services. Carbon dioxide has been captured and stored under Tomakomai Bay since 2016. As the first project of this size in Japan, extensive public communication and stakeholder engagement has been undertaken by project developer Japan CCS Company (JCCS). These activities have included annual public lectures on CCS in the city with guest speakers; guided tours of the CCS facilities; an educational outreach programme for schools; information boards in Tomakomai City Hall; provision of data from the carbon dioxide storage site online (see Table 3); and face-to-face meetings with stakeholders such as fishers (interview with JCCS). Whilst is not possible to assess whether such activities have made Tomakomai citizens more likely to engage with climate information services than their counterparts in other cities, the Tomakomai CCS Demonstration Project does illustrate how large-scale climate-related projects can provide an entry point for citizens to engage with climate information more broadly. For instance, JCCS’ Tomakomai Monitoring webpage (http://www.jccs-tomakomai-monitoring.com/JCCS/index.php/top/) also holds data on seasonally-observed carbon dioxide concentrations in Tomakomai Bay; and the 2019 public information lecture on CCS in Tomakomai incorporated a talk on climate change and its future effects given by Japanese mountaineer Ken Noguchi (Tomakomai City, 2019). Governments and researchers tasked with implementing public-facing climate information services may therefore wish to consider whether there are existing climate-related initiatives within the community with which citizens are already engaging, where additional climate-related data may gain more traction.

A second linked insight from Tomakomai concerns engagement with key stakeholder groups – in particular fishers. Engagement of fishers on climate change issues is particularly important in Tomakomai due to the social and cultural significance of Sakhalin surf clam fishing to the locale, which means protection of fisheries is not only an economic matter but also one of local pride and identity (Mabon et al., 2017). However, previous experiences of pollution of the marine environment negatively affecting fish stocks (first with discharges of pulp from a nearby paper factory in the 1920s–50s, then with disposal of material excavated during the Tomakomai port expansion in the 1960s–70s) has made fishers cautious – if not hostile – to engagement with external bodies on environmental issues (interview with fisheries cooperative). The support of fishers is especially important for deployment of the CCS initiative under Tomakomai Bay, given the potential of the development to contribute to climate change mitigation. In response, the Business Location Promotion Division of Tomakomai City Government and also research scientists surveying the marine environment of Tomakomai Bay have undertaken an extensive campaign of face-to-face engagement with the local fisheries cooperative. The purpose of doing so is to build and maintain fishers' support for the CCS demonstration project by explaining the results of marine survey and monitoring data taken around the storage site, and to discuss climate changes and their effects on fisheries more widely at the same time (interview with Tomakomai City Government Business Location Promotion Division). Fishers in Tomakomai are indeed concerned about the effects of climate change on fish stocks and are keen for data to help understand what it means for them (interview with fisheries cooperative), hence this illustrates a case where face-to-face communication and explanation through key trusted sources helps to back up the hard data gleaned through monitoring of the marine environment.

A third area of challenge identified within the Tomakomai research concerns the need to connect climate information services underpinned with environmental data on one hand, with the embodied and anecdotal ways in which people experience changes in the environment on the other. For instance, interviewed fishers reported that different fish were being caught than previously, tying these observations to a changing climate (interview with fisheries cooperative). A regional environmental NGO representative likewise indicated that different species were being found within Hokkaido, and that seasons and weather patterns were changing – however, he noted that such observations were based on people's observations, sensations and feelings, and not necessarily on empirical data (interview with regional environmental NGO). There was nothing in the interview data to indicate that the climate information services available within Tomakomai City in any way contradict the personal and embodied experiences of interviewees. Yet these points nonetheless demonstrate that citizens may come to engage with climate issues – and hence form their views on climate risks and how to respond - not through techno-scientific data, but rather through experiences in their everyday lives.

Fourth and related is the challenge which emerged in the Tomakomai interviews around engaging with citizens and peripheral stakeholders in locations where a changing climate may be seen as a positive. Given the cold climate in southern Hokkaido, interviewees in some cases made flippant if well-intentioned remarks about how a warming climate could bring benefit to the locality. The examples of Hokkaido rice becoming more delicious as a result of rising average temperatures, or warmer winters leading to less troublesome snowfalls were, for instance, raised in informal conversations with citizens. In other cases, climate information (i.e. long-term temperature and ice predictions) fuelled a perception that a changing climate could bring localised economic benefit to Hokkaido by allowing the city's port to capitalise on opening trade routes through melting Arctic sea ice (interview with port authority; interview with city chamber of commerce^1^). In the more rural periphery around Tomakomai, the limited contribution of the locale to Japan's carbon dioxide emissions was used as an explanation for limited engagement with climate change data and knowledge more widely (interview with urban planner). All of the above indicate that in locations which may not be perceived to be at immediate risk from changes in the local climate, as per Section 2.2. there may be a need to tailor the framing and messaging of climate information services towards the negative effects of climate change more widely as well as less well-understood risks for the immediate locality. It is perhaps for this reason that Tomakomai City's public-facing summary of their climate change action plan (Tomakomai City, 2013) places heavy emphasis on visualisation of the changes to the natural environment which may be caused through climate change, providing photographic evidence of shrinking apples and dying trees in landscapes similar to those found in Tomakomai.

## 6 Discussion and conclusions

The paper finishes with an extended conclusion, which makes recommendations for policy- and decision-makers responsible for implementing public-facing climate information services at the city level. Each recommendation is discussed with reference to field observations from Fukuoka and Tomakomai, and where appropriate through links back to the literature review in Section 2.

The first recommendation is that urban climate policy-makers should not underestimate citizens' and peripheral stakeholders' awareness of climate change issues, or their willingness to engage with potentially complex data. It is of course true that ‘translating’ knowledge and enhancing user capacity to understand complicated data are recognised challenges for making public-facing climate information services more effective (McNie, 2013). Both Fukuoka and Tomakomai have sought to undertake such ‘translation’ work through, for example, the provision of hazard maps showing areas of risk on maps of the city alongside preparation and evacuation information. Equally, however, the examples of community climate volunteers in Fukuoka attending training sessions and academic lectures to boost their knowledge of local climate change effects (Section 4) and fishers' acknowledgement of the need for data to understand climate change in Tomakomai (Section 5) illustrate it is not necessarily the case that publics need to be ‘informed’ or ‘convinced’ about urban climate risk. Reinforcing the argument of Carr et al. (2013) in the context of geoengineering and the Kasperson (2014) advocacy for avoiding assumption about what user needs will be in risk communication, the Fukuoka and Tomakomai findings suggest that when it comes to climate information services, publics can be ready to engage with issues of significant complexity on climate change. It is however valuable for there to be a clear pathway for informed citizens and stakeholders to access techno-scientific experts within the city who can explain complex data to them face-to-face, similar to the Kyushu Environmental Evaluation Association/Fukuoka Center for Climate Change Actions in Fukuoka and the Business Location Promotion Division and collaborating marine scientists in Tomakomai. As these informed citizens may go on to be trusted points of contact and opinion-shapers in their own communities, it is important that relevant experts are given the time and resources to engage meaningfully with publics on urban climate information services.

The second recommendation is to respect that people have their own lived experiences of climate change, which inform how they engage with climate information services and how they perceive data and information. People will not necessarily learn about urban climate change ‘scientifically’, rather they may start to develop interest in the issue through their own embodied and anecdotal examples, which can be a trigger for engaging with climate information services. This can be seen through the ways in which respondents in Tomakomai narrate climate change in terms of changes in species seen in the everyday environment, perceived frequency and extent of snowfall, and even the taste of local rice. In Fukuoka too, periods of hot weather and memories of recent severe rain events serve as analogues for a changing climate, as do everyday observations such as the temperatures of school playgrounds. Whilst it may be tempting to disregard such experiences as an ‘unscientific’ indicator of climate risk, Bradbury (1989) and Oughton (2013) both warn that dismissing outright concerns grounded in feelings or emotions risks alienating or marginalising publics in risk governance processes. Returning to the importance of environmental justice in creating a fully resilient city, recognition of different identities and knowledge systems is argued to form one component of urban climate justice (Reckien et al., 2018). As such, it could be argued that recognition of citizens' embodied experiences and knowledges within the governance of urban climate risk is an important part of creating an equitable and resilient city. Again, this does not mean ‘anything goes’. Rather, all it means is that local government climate managers and researchers providing data ought not to view the role of climate information systems as being to ‘correct’ misunderstandings that people may have. Climate information systems – and their providers – hence should be able to facilitate respectful dialogue with publics on how their personal experiences of environmental change in the built environment may match up to the trends and patterns observed in techno-scientific data.

The third recommendation is for risk-managers (in this context, local government divisions tasked with hosting and implementing urban climate information systems, and universities, research institutes and civil society organisations who may also provide public-facing information on urban climate) to recognise that the institution, organisation or person who is a ‘trusted’ source of information may not be the institution, organisation or person responsible for climate governance in a city. In other words, the people that citizens trust and look to for information to help them make decisions about climate change risk in their lived environment might not be the people closest to setting urban climate policy or hosting climate information services. In Tomakomai, for example, the Business Location Promotion Division of the city government – not always the environment division – are a main point of contact on climate change with the fishing cooperatives, largely due to the Business Location Promotion Division's leadership on the city's carbon dioxide capture and storage project which has entailed extensive consultation with fishers. In Fukuoka, academic leadership on climate change action has over the last decades come not from a natural scientist, but from a scholar of environmental law in Professor Naohito Asano of Fukuoka University due to his expertise in environmental protection and urban environmental planning laws. Similarly in Fukuoka, for heat risk in particular the city government's greenspace division have a big role to play in knowledge-sharing around cooling, yet they are not connected to climate action specifically. Reflecting the idea of risk governance as a dialogic process (van Asselt and Renn, 2011), during the development of public-facing climate information services, there is hence a need to understand who exactly – and why – citizens look to for information to help them make decisions on climate risk. There is a concomitant need for knowledge- and data-sharing across sectors and institutions, so that those being contacted by citizens for climate risk guidance are able to access the underlying climate data and information.

## Declaration of Competing Interest

None.

## Appendix A Supplementary data

Supplementary material Image 1

## Footnotes

1 To be fair to the respondents, it is important to acknowledge that in both cases the interviewees did elsewhere in their interviews discuss the severity of the climate challenge to Japan and the need to take action within Tomakomai.
