# Supplementary material for: Making climate information services accessible to communities: What can we learn from environmental risk communication research?
Source: Urban Clim. 2020 Mar;31:100537. doi: 10.1016/j.uclim.2019.100537 (PMC7043330; doi:10.1016/j.uclim.2019.100537)
Supplement: Supplementary file 1 — Supplementary material [file mmc1.pdf]

MAKING CLIMATE INFORMATION SERVICES ACCESSIBLE TO COMMUNITIES: WHAT CAN WE LEARN FROM ENVIRONMENTAL RISK COMMUNICATION RESEARCH?

SUPPLEMENTARY DATA

(a) Sample interview schedule for Fukuoka interviews

1. What are the effects of climate change from the perspective of Fukuoka?

(a) What are the main effects of climate change in Fukuoka?

(b) What do you think the risks and effects are on Fukuoka's citizens from climate change?

(c) What is the biggest concern about climate change at present from the Fukuoka level?

(d) Are there existing/current effects on environment or lifestyle from climate change, for:
   a. Weather?
   b. Landscape?
   c. Etc?

(e) What activities are you undertaking to increase citizens' awareness of climate change within Fukuoka?

2. Tell me about climate change governance and policy at the Fukuoka administrative level

(a) What are the processes for creating environment and climate change visions and policies in Fukuoka?
   a. Participation of experts?
   b. Stakeholder engagement?
   c. Citizen engagement?

(b) How do these strategies and policies link to other climate and environment policies, specifically:
   a. Smaller spatial scales (e.g. municipal/neighbourhood)?
   b. Larger spatial scales (e.g. national)?

(c) What kinds of international knowledge exchange on climate adaptation does Fukuoka participate in?
   a. With which cities and countries?
   b. What are the benefits to Fukuoka?

(d) What are the next steps for development of climate and environment policy?
   a. Tell me about the revision and development process;
   b. What is the timeframe for revised versions of existing policies and strategies?

(b) Sample interview schedule for Tomakomai interviews (see also Mabon et al, 2017)

1. Context and history

(a) Tell me about your organisation
- (i) When was it founded?
- (ii) What is its purpose?
- (iii) How does it relate to the environment and climate change?
- (iv) etc

(b) Tell me about the current social situation of the city (Tomakomai/Muroran/Yubari etc)
- (i) Employment base?
- (ii) General economic situation?
- (iii) Cultural situation/activity?

(c) Tell me about the history of the city (Tomakomai/Muroran/Yubari etc)
- (i) How has the economic and employment base changed over time?
- (ii) How has the city expanded/developed over time?
- (iii) How do you think society and culture has changed over time in the city?

2. Environment and climate issues

(a) What environmental issues are you/your organisation facing at the moment?
- (i) How have things changed in the last 10-20 years?
- (ii) Are there any issues that are going to become a bigger problem into the future?

(b) How does climate change fit into these?
- (i) How has the climate changed in the last 10-20 years?
- (ii) What effects do you expect to see from now into the future?

(c) What policies or countermeasures are you/your organisation taking against climate change?
- (i) Regulations?
- (ii) Policies?
- (iii) Anything you are especially concerned about?

3. CCS and low-carbon energy infrastructure

(a) Tell me what you know about CCS?

(b) Based on what you know, what benefits do you think CCS could bring:
- (i) To your organisation;
- (ii) To this region (e.g. hotels, income etc)?
- (iii) To Japan in general?

(c) What other energy or large infrastructure projects are going on in the area just now?
- (i) How do you think these might affect your organisation?
- (ii) How do you think the community feels about them? Why?
